# TarTar: A Timed Automata Repair Tool

**DOI:** 10.1007/978-3-030-53288-8_25

**Published:** 2020-06-13

**Authors:** Martin Kölbl, Stefan Leue, Thomas Wies

**Affiliations:** 8 Microsoft Research Lab, Redmond, WA USA; 9 University of Southern California, Los Angeles, CA USA; 10 University of Konstanz, Konstanz, Germany; 11 New York University, New York, USA

## Abstract

We present TarTar, an automatic repair analysis tool that, given a timed diagnostic trace (TDT) obtained during the model checking of a timed automaton model, suggests possible syntactic repairs of the analyzed model. The suggested repairs include modified values for clock bounds in location invariants and transition guards, adding or removing clock resets, etc. The proposed repairs guarantee that the given TDT is no longer feasible in the repaired model, while preserving the overall functional behavior of the system. We give insights into the design and architecture of TarTar, and show that it can successfully repair 69% of the seeded errors in system models taken from a diverse suite of case studies.

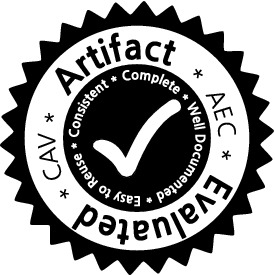



## Introduction

A reactive system with requirements pertaining to its timing behavior is often modeled as a network of timed automata (NTA) [BY03]. Whether a timing requirement holds in an NTA can be analyzed by timed model checkers such as Uppaal [BLL+95] or opaal [DHJ+11]. In case of a requirement violation, a model checker returns a timed counterexample, also called a timed diagnostic trace (TDT). Until now, developers must manually identify and correct such violations by analyzing the generated TDTs. It is therefore desirable to support this process by an automated tool set that not only determines whether timing requirements are met, but also proposes syntactic repairs of the NTA in case they are not.

In [KLW19] we presented an automated repair analysis that analyzes a TDT obtained from the violation of a timed safety property and returns syntactic repair suggestions that avoid the concrete executions of the TDT violating the property. The analysis performs an additional admissibility check ensuring that the repaired model is functionally equivalent with the original NTA, which means that no action traces are added or omitted by the repair.

**Fig. 1.**
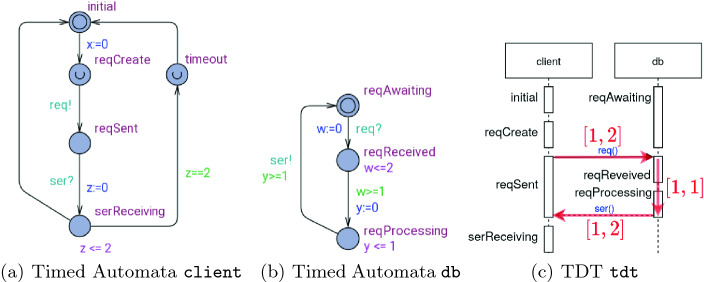
Network of timed automata - running example

To illustrate the repair analysis consider the NTA in Figs. 1(a) and (b). It describes a *client* that sends a request *req* to a database *db* and expects to receive a response *ser* within 4 time units after sending the request. The client contains a clock *x* that measures the time delay between the request creation and the receiving of a response in location *serReceiving*. The NTA allows to execute a TDT that violates the property, illustrated as a sequence diagram with time intervals in Fig. 1(c). A time interval in the sequence diagram denotes the minimal and maximal time delay for the message transmission and processing times in *db*, respectively. The repair computation analyzes the TDT and produces several syntactic repairs to the NTA that avoid the property violation. In [KLW19], the computed repairs aim at the modification of clock bounds in location invariants and transition guards. An example of such a repair is to reduce the bound in the time constraint $w \le 2$ from 2 to 1. The modified bound constrains the maximal transmit time of the *req* message so that the resulting NTA receives all responses within the expected time. This repair eliminates the problematic executions of the TDT in the original NTA without changing the functional behavior of the system, which is confirmed by an admissibility test defined in [KLW19]. However, in general, it may not be possible to repair the model using only clock bound alterations.

*Contributions.* We present TarTar [tar20], which extends the initial prototype implementation of the clock bound repair analysis presented in [KLW19] to a more comprehensive NTA repair tool. Specifically, the extended tool implements new analyses that can suggest a whole range of repairs in addition to clock bound variation, such as modifying comparison operators in constraints, clock references, clock resets, and location urgency. Examples of new repairs computed for the model in Fig. 1 are:

- Exchanging the comparison operator in the constraint $w \ge 1$ to $w<1$ ensures that the time to send a request is below 1 time unit.
- An exchange of clock *z* in $z \le 2$ with clock *y* restricts the time of processing and receiving the response to at most 2 time units.
- To reset the clock *y* on the previous transition instead ensures that the time for sending and processing the request is below 1 time unit.
- Making the location *serReceiving* urgent reduces the time to receive a response to 0.

We call a repair admissible if the repaired system is functionally equivalent to the unrepaired system. The repair analysis implemented in TarTar returns the complete set of admissible repairs.

The repair analysis combines concepts and algorithms from model checking, constraint solving, and automata theory. A real-time model checker is used to generate TDTs for a given NTA that violate a given timed safety property. TarTar translates the TDT into a linear real arithmetic constraint system. An SMT solver is used to compute a repair for the generated constraint system by solving a MaxSMT problem. An automata-based language equivalence test checks whether the repair is admissible in the NTA model. The collaboration between these subcomponents yields a complex tool architecture. We provide insights into the design and implementation of this architecture and the underlying infrastructure of supporting tools. We evaluate the new repair analyses by applying TarTar to a number of NTA models. We systematically inject different modifications in these correct models and compute repairs for the obtained faulty models, which results in at least one admissible repair for 69% of the TDTs.

*Related Work.* Other tools exist that compute repairs. The tool BugAssist [JM11] analyzes C-code by solving a MaxSMT problem. The tool ReAssert [DDG+11] checks a set of possible modification to repair broken unit tests. Angelix [MYR16], S3 [LCL+17] and SemFix [NQRC13] compute repairs by symbolic execution and constraint solving. SketchFix [HZWK18] is based on lazy candidate generation. All tools are not repairing broken time constraints. We are not aware of related work on tools for the repair of timed automata models. A more comprehensive overview of related work on automated repair is given in [LPR19]. A discussion of work related to the foundations of our repair analysis can be found in [KLW19].

## New Types of Repair Analyses

The repair analysis presented in [KLW19] and implemented in the prototype version of TarTar encodes a TDT as a constraint system in linear real arithmetic. It computes syntactic correct modifications of the underlying NTA by introducing bound variation variables $$ v $$. For example, possible bound modifications for a clock bound $$x \le 2$$ are expressed by a modified clock bound $$x\le 2+ v $$. The repairs are computed by solving a partial SMT problem on the TDT constraint system, involving soft-assert constraints on the bound variation variables. No repair is computed whenever the soft assertion $$ v =0$$ holds, otherwise the computed value of $$ v $$ characterizes the repair. In the following we sketch the new types of repairs implemented in TarTar. For a more comprehensive description, which space limitations do not allow us to provide here, we refer to [KLW20].

*Operator Variation Repair Analysis.* This analysis is motivated by the assumption that a wrong comparison operator in a location invariant or transition guard may cause a property violation. We assume for the repair encoding that the operators $$\sim $$ are indexed according to their order in the sequence $$\langle \; <, \le , =, \ge ,>\> \rangle $$. The possible repairs are encoded by a fresh variation variable $$ v ^{ov}_i$$ where the value of $$ v ^{ov}_i$$ is the index of the corresponding comparison operator. If $$x < 4$$ is computed as a repair, then $$ v ^{ov}_i= 1$$.    Using this repair analysis, TarTar finds two admissible repairs for the example in Figs. 1(a) and (b) that replace the comparison operator in the clock constraint $$w>=1$$ by < or $$<=$$, respectively.

*Clock Reference Repair Analysis.* This analysis aims to repair property violations resulting from errors that stem from the unintended use of a wrong clock variable. We enumerate all the positions of clock variables in clock bound constraints using index $i$ and all clock variables using index *k*. We then introduce for every position $i$, a fresh variation variable $v ^{{\textit{cv}}}_i$ whose value *k* indicates the clock $c_k$ to be used at that position in the repaired model. For example, if $y \le 2$ is a repaired constraint, where the position of *y* in the constraint has index 3 and clock *y* has index 1, then $v ^{{\textit{cv}}}_3 = 1$. Applying this repair analysis to the examples in Figs. 1(a) and (b), TarTar finds 13 admissible clock reference modification repairs, each involving two modifications. Nine repairs exchange *y* in the constraints $y \le 1$ and $y \ge 1$ by a selection from the set of clocks *z*, *x* and *w*. Four repairs exchange *y* in the constraint $y \le 1$ by *w* or *x*, and *w* in the constraint $w\ge 1$ by *y* or *z*.

*Reset Clock Repair Analysis.* This analysis aims to repair a property violation by adding or removing clock resets. We introduce a variation variable $v ^{{\textit{rv}}}_{i,j}$ for each clock $c_i$ and the transition leaving location $\lambda _j$ in the TDT. The reset status in the extended constraint system is inverted when $v ^{{\textit{rv}}}_{i,j} \ne 0$: if $c_i$ was not reset before, it will now be reset, and vice versa. Applying the reset repair analysis to the examples in Figs. 1(a) and (b), TarTar finds four admissible repairs. One repair removes the reset of clock *y*, another removes the reset of clock *z* and two repairs add a reset of clock *x* either on the transitions towards the state *reqProcessing* or the transition towards the state *serReceiving*.

*Urgent Location Repair Analysis.* This analysis aims to repair cases where a faulty usage of urgent locations, which are always left with zero delay after entering, causes a property violation. Urgency of a location is modeled in the TDT constraint system by setting the location delay $\delta _j$ to 0. We define a fresh variation variable $v ^{{\textit{uv}}}_i$ for a location $\lambda _j$. For $v ^{{\textit{uv}}}_i\not = 0$, the urgency for a location $\lambda _j$ is inverted.    Applying the urgency location repair analysis to the examples in Figs. 1(a) and (b), TarTar finds two inadmissible repairs. The first one makes the state *reqAwaiting* urgent, and another repair makes the state *serReceiving* urgent.

## Usage of TarTar

We have implemented all repair analyses described in [KLW19] and in this paper in a tool named TarTar. It provides a graphical user interface, a command-line interface and a web-interface which enables the execution of this resource intensive software on compute servers. A user selects one of these interfaces via arguments provided when invoking the Java library implementing TarTar. For real-time model checking, TarTar relies on Uppaal.

**Fig. 2.**
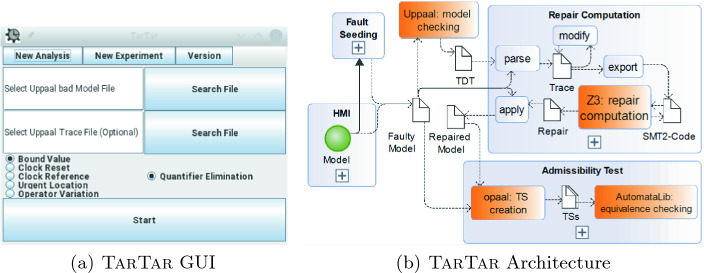
TarTar tool

- The argument *–web* launches the web server and corresponding interface.
- Any other arguments launches the command-line mode. When using the argument *–help*, the command-line console prints some help information.
- When no arguments are given, the graphical user interface depicted in Fig. 2(a) is launched. The interface offers three tabs. *New Analysis* starts a repair analysis, *New Experiment* starts fault seeding which is described later in Sect. 5, and *Version* shows the current version number of TarTar.

All tool interfaces expect the same types of inputs in order to start a TarTar analysis run. The user specifies a file containing the Uppaal model as input and selects the kind of repair to compute. Optionally, a file with a TDT of the given Uppaal model can be specified. When no TDT is provided, TarTar automatically calls Uppaal to compute a TDT. The result of an analysis is one repaired model file for every computed repair, as well as a text file that summarizes which repairs are admissible.

## Software Architecture and Implementation of TarTar

The software architecture of TarTar is depicted in Fig. 2(b). The orange rectangles in the figure represent external tools that TarTar calls in the course of the repair analysis. Uppaal is a state-of-the-art and closed-source model checking tool, which TarTar uses to compute a TDT for a given model and property. The SMT solver Z3 [dMB08] is used to solve the generated partial MaxSMT problems. To check the admissibility of a repair, TarTar uses opaal and the AutomataLib component of LearnLib [IHS15] since they conveniently provide functionality used during admissibility checking.

*Data Flow Architecture.* TarTar consists of many computation steps. For example, a TDT is parsed internally and stored as a Trace. This Trace is then modified and exported as SMT-LIB2 [BFT17] code. We define a computation step of TarTar as the computation transforming input into result artifacts. This focus on artifacts ensures a highly cohesive architecture and clear interfaces between any two computation steps. Computation steps with identical objectives are grouped into a project. This results in four projects depicted by blue rectangles in Fig. 2(b).

- *HMI* denotes the user interfaces of TarTar. The user inputs a timed model. TarTar then calls the project *Repair Computation* using a faulty timed model as a parameter. In case that the model is correct, TarTar calls the project *Fault Seeding*.
- *Fault Seeding* seeds faults into a correct model and then repairs the faulty model by computing repairs using *Repair Computation*. We use this analysis in Sect. 5 in order to benchmark the *Repair Computation* analyses.
- *Repair Computation* computes candidate repairs for a faulty timed model, applies these repairs to the model and finally automatically calls the *Admissibility Test*.
- *Admissibility Test* checks for every repaired model whether the computed repair is also admissible.

*Control Flow Architecture.* TarTar computes iteratively a set of repairs for a given faulty Uppaal model and a given property $\varPi$ using the following steps:

0. *Counterexample Creation*. TarTar calls Uppaal to verify the model against $\varPi$. In case $\varPi$ is violated, it stores a shortest symbolic TDT witnessing the violation in XML format.
1. *Diagnostic Trace Creation*. TarTar parses the model and the TDT into a data structure *Trace*. To add potential repairs, TarTar copies the trace and replaces the constraints that will potentially be subject to a repair by their modified variants. The modified trace is then translated to a logic constraint system, represented in SMT-LIB2 code.
2. *Repair Computation*. Z3 [dMB08] then solves a MaxSMT problem on the modified trace constraint system, computing a repair in which the number of unmodified constraints on the variation variables of type $$ v =0$$ is maximized. Since Z3 can solve a MaxSMT problem only for quantifier-free linear real arithmetic, TarTar first runs a quantifier elimination on the constraint system. It then solves the MaxSMT problem with soft constraints requiring $$ v =0$$ for all variation variables. For a more comprehensive presentation of this construction we refer the reader to [KLW20]. In case no solution is found, TarTar terminates. Otherwise, TarTar applies the repair to the faulty model and returns a repaired model.
3. *Admissibility Check*. TarTar checks the admissibility of a repair and compares the untimed languages of the faulty and repaired models. TarTar calls the model checker opaal in order to compute the timed transition systems (TTS) of the original and the repaired Uppaal model. We modified the opaal model checker in such a way that it returns the TTS for a model. TarTar then checks whether the two TTS have equivalent untimed languages, in which case the repair is admissible. This check is implemented using the library AutomataLib. In case the two TTS are not equivalent, the admissibility test returns a trace as a witness for the difference.
4. *Iteration*. TarTar enumerates all repairs, i.e., all combinations of constraint modifications that correct the TDT. The repairs are iteratively enumerated starting with the ones that require the smallest number of modifications to the model. After a repair is computed, the combination of modified variables that has been found is prevented from being reconsidered for future repairs by setting these modification variables to 0 using hard asserts. TarTar then proceeds with attempting to compute further, previously unconsidered repairs.

**Fig. 3.**
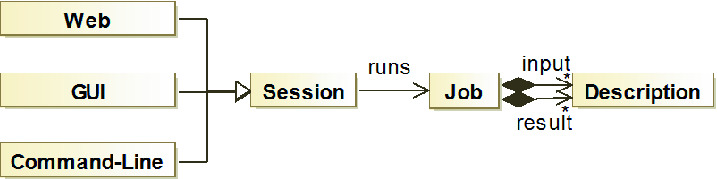
TarTar component architecture

*Component Architecture.* We implemented TarTar with the general infrastructure depicted in Fig. 3. The interface *Job* provides a general abstraction for an algorithm and specifies the necessary input and result values of the algorithm by the class *Description*. TarTar contains a *Job* for the projects *Fault Seeding*, *Repair Computations* and *Admissibility Test*. The class *Session* executes a *Job* and derivations of *Session* provide the different interfaces to the user. With this infrastructure, the analysis implementation in TarTar is independent from the implementation of the user interfaces, thus reducing coupling and improving modifiability of the code.

*Implementation Details.* We implemented the different projects that constitute TarTar in Java and use the build-management tool maven [Mav19] to manage the dependencies between the projects. TarTar interacts differently with the external tools that are needed for different purposes. It calls Uppaal via the command-line interface in order to generate a TDT and calls Z3 via its API to compute a repair. For the admissibility check, it calls opaal using a command-line script and the AutomataLib as an included Java library. For the implementation of the TarTar analyses the following two details are essential.

We modify constraints in an Uppaal model in order to apply a repair or to seed a fault. Since neither clock constraints nor transitions possess explicit unique identifiers in an Uppaal model, it is not obvious which constraint to change. We therefore uniquely identify a constraint by traversing the constraints in the sequence stored in the Uppaal model file and use the constraint index in this sequence as its identifier.

The complexity of the algorithms for solving quantifier elimination and the MaxSMT problem increase exponentially with the number of variables in the SMT model [KLW19]. We therefore reduce the number of variables by exploiting implied equality constraints. For example, a variable $c_j$ is created for every clock *c* in every step *j* of the TDT. We eliminate $c_j$ explicitly before quantifier elimination by replacing it with the term $\sum \nolimits _{i \in r..j}d_i$, where $d_i$ is the time delay at step *i* in the trace and *r* is the last step before *j* where *c* was reset.

## Evaluation

*Evaluation Strategy.* In order to evaluate the repair analyses both qualitatively and quantitatively, we need to synthesize a set of faulty timed automata. To the best of our knowledge, no benchmark suite for faulty timed automata exists. We therefore create faulty models by using the fault seeding strategy from [KLW19] which is motivated by ideas from mutation testing [JH11]. Mutation testing evaluates the quality of a test suite for a given program by systematically corrupting program code and determining the ratio of corruptions that the test suite is able to detect. We apply the same principle to evaluate the quality of our repair technique. As proposed in [KLW19], fault seeding modifies a single clock constraint so that the result is a set of models that violate a given property. During the seeding, the bound of a single clock constraint is modified by an amount of $\{-10, -1, +1, +0.1M, +M\}$, where M is the maximal clock bound occurring in a given model. Our observation was that making either small modifications that are close to the bound value or modifications in the order of the maximal bound value M often introduce actual errors in the model. We have extended fault seeding to the new types of repairs. In particular, fault seeding additionally exchanges the comparison operator in a clock constraint by $\{<, \le , =, \ge , >\}$, swap a referenced clock with all other clocks occurring in the given model, modify the reset clocks of any transition, and switch for any location whether it is urgent. TarTar checks automatically whether a modified TA violates a given property. If this is the case, it performs all of the above defined repair analyses.

*Results.* We applied fault seeding to the models in [KLW19] and analyzed the obtained TDTs using the above described repair analyses implemented in TarTar. All analyses were performed on a computer with an i7-6700K CPU (4.00 GHz), 60 GB of RAM and a 64 bit Linux operating system. We summarize the results of the experiment per considered model (Table 1) and per type of considered repair (Table 2). Column *Sd* contains the count of seeded faults that result in a number *#T* of faulty models. $T_{\textit{UP}}$ is the maximal time that Uppaal needs to create a TDT for the faulty models, and the longest TDT has a length of *Ln*. TarTar computed for the TDTs overall a number #*R* repairs of which #*A* are admissible. An admissible repair is found for #*S* of the TDTs. The computation effort for a repair analysis is given by the time $T_{\textit{QE}}$ for successful quantifier elimination, the number of timeouts #*O* of quantifier eliminations after 10 min, the average time $T_{\textit{R}}$ to compute a repair and the memory consumption $M_R$. The constraint system that Z3 solves has the count #*Vr* of variables and #*Cn* of constraints. The effort for the admissibility check is given in time $T_{\textit{Adm}}$ and memory $M_A$. All times are given in seconds and memory consumption in MB. Notice that we omit the columns pertaining to the fault seeding and TDT computation in Table 2 as they are irrelevant here.

**Table 1.** Experimental results according to model.

Repair	#Sd	#T	$T_{\textit{UP}}$	*Ln*	*#R*	*#A*	*#S*	$T_{\textit{QE}}$	#O	$T_{\textit{R}}$	$M_R$	*#Vr*	*#Cn*	$T_{\textit{Adm}}$	$M_A$
db rep.	110	13	0.016	4	229	138	9	89.346	2	0.911	14.53	30	91	2.080	45
csma	191	10	0.012	2	70	26	8	0.049	0	0.023	0.58	16	72	1.825	75
elevator	88	5	0.011	1	7	5	4	0.049	0	0.020	0.53	6	28	1.665	17
viking	310	9	0.015	18	9	7	5	86.539	21	1.436	20.07	120	180	1.952	543
bando	1,955	40	0.111	279	4,061	209	21	31.555	46	4.922	20.86	1,156	8,144	19.57	1251
Pacemaker	1,187	12	0.022	9	62	19	10	0.663	20	0.325	2.59	116	988	1.994	206
SBR	353	50	0.027	84	751	660	31	117.057	86	2.686	37.16	765	1,211	138.004	211
FDDI	314	36	0.014	11	166	105	34	29.859	51	3.074	9.70	116	272	2.241	128

Overall, TarTar seeded 4.508 faults. This resulted in 175 TDTs in total (60 TDTs due to bound modification, 72 due to operator variation, 27 due to changing the clock reference, 8 due to complementing the reset of clocks and 8 due to the switching of urgent locations). TarTar found 5,355 repairs, out of which 1,169 were admissible. It found at least one admissible repair for 122 of the TDTs. The maximal number of modified constraints in the admissible repairs computed for a single TDT using all types of analysis was 25.

**Table 2.** Experimental results according to type of repair.

Repair	*#R*	*#A*	*#S*	$T_{\textit{QE}}$	#O	$T_{\textit{R}}$	$M_R$	*#Vr*	*#Cn*	$T_{\textit{Adm}}$	$M_A$
Bound Modification	533	364	85	15.209	8	4.922	20.86	1,156	2,498	138.004	525
Operator Variation	3,929	96	51	117.057	44	2.686	37.16	996	8,144	59.117	543
Clock Reference	693	625	35	33.282	61	3.074	14.13	1,120	5,355	116.944	206
Reset Clock	45	37	13	89.346	113	0.911	14.53	996	2,836	2.051	45
Urgent Location	155	47	37	0.107	0	0.135	3.16	1,120	2,502	58.551	1,251

*Interpretation.* Few of the seeded faults resulted in a property violation. TarTar seeded 4.508 faults which led to 175 TDTs, thus only 3.9% of these faults result in a TDT. This supports the hypothesis that, in practice, often times only few time constraints have an impact on a property violation. TarTar computes at least one admissible repair by bound modification for 85 (48%) of the 175 TDTs, by operator variation for 51 (29%), by clock reference for 35 (20%), by clock reset for 13 (7%) and by urgent location for 37 (21%). Every analysis on its own computes less admissible repairs than the combination of all repair analyses, which solves 122 (69%) of the 175 TDTs. The largest number of modified constraints in all the admissible repairs for a single TDT was 25, which is less than anticipated. This low number of modified constraints infer that, for the examples that we considered, only a few constraints of each TDT combined to admissible repairs. The number of modified constraints determines the number of possible repairs that have an impact on whether a property is violated or not. Since it was observed in [KLW19] that the computational effort for the repair computation is largely determined by the quantifier elimination step, we expect that in light of the observed 226 timeouts a more efficient quantifier elimination would lead to a significantly higher number of repairs. Furthermore, the number of timeouts, and thus the computation time needed for the repair, rises with the length of the analyzed TDT. The model *SBR* has the most timeouts with 86 and the third longest trace with a length of 84 steps. The model *bando* has the third most timeouts with 46 and the longest trace. Obviously, the longer the TDT, the larger the resulting constraint system, leading to increased computational effort. The *bando* model has the largest constraint system with 1, 156 variables and 8, 144 constraints. The *SBR* model has the second largest constraint system with 765 variables and 1, 211 constraints. The model *FDDI* has a shorter trace of length of 11 and a much smaller constraint system with 116 variables and 272 constraints. From this we conclude that the complexity of a repair depends not only on the trace length, but also on the intrinsic complexity of the model. Modifying states from urgent to non-urgent during fault seeding resulted in only 8 TDTs. This low number is due to the observation that the considered models contain only few urgent states. Modifying non-urgent states to urgent ones, however, did not lead to a single property violation resulting in a TDT. The rationale is that urgency ensures to leave a state immediately without a delay which leads to a restriction rather than a relaxation regarding the time budget spent along an execution trace. As a consequence, making a state urgent does not cause a property violation in many models since the type of the checked properties is typically time bounded reachability, and a restricted time budget does not make it more likely that the property is violated. We finally observe that the admissibility check requires more computation resources than the repair computation. The maximal memory used for the admissibility test was $1,251\,\text {MB}$ in contrast to $37.16\,\text {MB}$ for the repair computation. This is in line with our expectation since the admissibility test searches the state space of the full NTA, while the repair analyses only considers a single TDT.

## Conclusion

We have presented the TarTar tool, its architecture and implementation, and illustrated its application to a number of significant case studies. In the course of our work we have extended the repair analysis that is implemented in TarTar for bound modification to modifications of comparison operators, clock references, reset of clocks and missing urgencies. The evaluation of the repair analyses showed that an admissible repair is computed for at least 69% of the analyzed TDTs. The integration of various tools with heterogeneous interfaces posed a particular challenge to the architecture of TarTar which we addressed by the definition of intermediate artifacts.

In future work we plan to explore the interplay between different repairs that are computed for a repaired system that still violates a property, and develop refined strategies to select promising repairs from a repair set. A further generalization of the analysis is to not only compute clock constraint modifications for faulty models but also to compute possible relaxations of clock constraints for correct models in order to support design space exploration.
